# Overexpression of Glucocorticoid-induced Leucine Zipper (GILZ) increases susceptibility to Imiquimod-induced psoriasis and involves cutaneous activation of TGF-β1

**DOI:** 10.1038/srep38825

**Published:** 2016-12-09

**Authors:** Elena Carceller, Marlies Ballegeer, Julie Deckers, Carlo Riccardi, Stefano Bruscoli, Tino Hochepied, Claude Libert, Paloma Pérez

**Affiliations:** 1 Instituto de Biomedicina de Valencia-Consejo Superior de Investigaciones Científicas (IBV-CSIC), Jaime Roig 11, E-46010 Valencia, Spain; 2 Inflammation Research Center, VIB, B-9052 Ghent, Belgium; 3 Department of Biomedical Molecular Biology, Ghent University, B-9052 Ghent, Belgium; 4 Laboratory of Immunoregulation and Mucosal Immunology, Ghent University, B-9000 Ghent, Belgium; 5 Receptor Research Laboratories, Nuclear Receptor Lab, Medical Biotechnology Center Cytokine Receptor Laboratory, VIB, B-9000 Ghent, Belgium; 6 Department of Medicine, University of Perugia Medical School, 06132 Perugia, Italy

## Abstract

Psoriasis vulgaris is a chronic inflammatory skin disease affecting millions of people. Its pathophysiology is complex and involves a skin compartment with epidermal and immune cells which produce cytokines, e.g. belonging to the IL-23–Th17-cell axis. Glucocorticoids (GCs) are the most common therapeutics used in cutaneous inflammatory disorders and GC-induced leucine zipper (GILZ) has emerged as a mediator of GCs due to its anti-inflammatory actions, theoretically lacking GC side-effects. We evaluated whether GILZ may provide a better therapeutic index in comparison to GCs during the onset and progression of psoriasis by generating and characterizing a mouse model with generalized overexpression of this protein (GILZ-Tg mice) and the imiquimod (IMQ) psoriasis model. Unexpectedly, in GILZ-Tg mice, the severity of IMQ-induced psoriasis-like skin lesions as well as induction of cytokines commonly up-regulated in human psoriasis (*Il-17, Il-22, Il-23, Il-6, S100a8/a9*, and *Stat3*) was significantly more pronounced relative to GILZ-Wt mice. The increased susceptibility to IMQ-induced psoriasis of GILZ-Tg mice was significantly associated with skin-specific over-activation of TGF-β1-mediated signaling via SMAD2/3. Our findings demonstrate that GILZ may behave as pro-inflammatory protein in certain tissues and that, similar to prolonged GC therapy, GILZ as an alternative treatment for psoriasis may also have adverse effects.

Psoriasis vulgaris is a chronic relapsing inflammatory skin disease which affects 2–3% of the population and is characterized by demarcated red scaly skin lesions most often on the elbows, knees, hands and feet. The disease is characterized by hyperproliferation and abnormal differentiation of the epidermis along with infiltration of immune cells, mainly T cells and dendritic cells. Immune cells secrete high amounts of pro-inflammatory cytokines, which in turn induce production of additional cytokines by keratinocytes and other cutaneous cells^1^. In particular, T helper 17 (Th17) cells, activated and maintained by IL-23, secrete IL-17, IL-22 and TNFα, constituting the so-called IL-23–Th17-cell axis, which plays a major role in several diseases including psoriasis^2^,^3^,^4^.

The etiology of the disease is complex and includes defects in genes associated with the epidermal barrier (*Lce3* family members), NF-κB activation (*Rel, Nfkbia, Tnfaip3*), and adaptive immune responses (*Il-23r, Il-12b*), as well as alterations in different signaling pathways, as shown by studies in human patients and in mouse models^5^,^6^. In transgenic mice, keratinocyte-targeted overexpression of STAT3 and transforming growth factor-beta1 (TGF-β1) or combined deletion of JUN and JUNB in this cell type induces a pathology closely mimicking psoriatic skin^7^,^8^,^9^.

Synthetic GCs form the most common treatment for numerous cutaneous inflammatory and immune diseases including psoriasis, because of their anti-inflammatory potential^10^,^11^. However, the chronic use of GCs for treating psoriasis is compromised by adverse effects (skin atrophy, telangiectasia, and potential systemic detrimental actions), necessitating the search for more specific downstream mediators of GC action. GCs bind and activate the GC-receptor (GR) that acts as a ligand-dependent transcription factor, binding to hormone response elements or GREs at the regulatory sequences of its target genes, a process classically referred to as transactivation^12^,^13^. GR can also regulate gene expression by interfering with other transcription factors (e.g., NF-κB and AP-1) without direct binding to DNA (a process referred to as transrepression). It is currently accepted that both mechanisms of GR are required to achieve optimal anti-inflammatory GC effects^12^.

In the past few years, several anti-inflammatory mediators induced through GRE-dependent transcription, such as GC-induced leucine zipper (GILZ), have received increasing attention^13^,^14^,^15^. GILZ, which is encoded by the *Tsc22d3* gene, was originally described by the Riccardi laboratory as a dexamethasone-responsive gene in the thymus^16^ and is currently known to be ubiquitously expressed. The mouse and human *Tsc22d3* genes are highly similar at the nucleotide level (more than 90%) and encode a leucine zipper protein that modulates several signaling pathways, crucial to inflammation and immune response, including NF-κB and RAS/RAF/MAPK. GC-induced transcriptional up-regulation of GILZ interferes with NF-κB activation through direct interaction of GILZ with the p65 NF-κB protein, resulting in inhibition of NF-κB nuclear translocation, DNA binding, and transactivation^17^,^18^. In fact, GILZ has emerged as a possible alternative to GC therapies, due to its anti-inflammatory actions which are not accompanied by GC adverse effects, as demonstrated by several mouse models of inflammation and immune dysfunction^14^,^19^,^20^,^21^,^22^. High GILZ protein levels, by GILZ-overexpressing transgenic mice or by injection of TAT-GILZ fusion proteins, have shown to lead to diminished inflammatory responses in experimentally induced colitis. The effect was similar to dexamethasone, as quantitated by reduced pro-inflammatory Th1 cytokines, IFN-γ, and TNF-α^23^. Also, TAT-GILZ protected mice against LPS-induced endotoxemia^24^. An immunomodulatory GILZ-derived peptide ameliorated experimentally autoimmune encephalomyelitis^18^ and more recently it was reported that deficiency of GILZ in mice resulted in augmented inflammation after IMQ treatment, demonstrating that GILZ plays a T-cell intrinsic role limiting pathogenic Th17 responses in the context of psoriasis^25^. It must thus be concluded that in most experimental settings, GILZ appears as a key modulator of regulatory T cells, and constitutes a major mechanism of GC-mediated immunosuppression^14^,^22^,^26^,^27^.

We have previously reported that *Tsc22d3* is transcriptionally up-regulated during the differentiation of keratinocytes *in vitro*^28^; however, the role of GILZ in skin pathophysiology is largely unknown. Here, we aimed to evaluate the consequences of GILZ overexpression during the onset and progression of psoriasis by generating and characterizing a mouse model with generalized overexpression of this protein (GILZ-Tg mice). We describe the generation of these mice. Furthermore, we used the well-validated imiquimod (IMQ)-induced model of psoriasis in which psoriatic inflammation is induced by repetitive topical application of Aldara^®^, a cream containing 5% of the toll-like receptor 7 (TLR7)-agonist IMQ^29^,^30^. The phenotype of IMQ-treated mouse skin closely resembles the histological and molecular changes associated to human psoriasis, in particular the activation of the IL-23–Th17-cell axis. The key evidence supporting that IMQ treatment in mice is a relevant model for studying human psoriasis is that the clinical use of Aldara^®^ for treating cutaneous keratosis, superficial *basal cell carcinoma and warts* produces local reddening and inflammation in all treated patients^29^,^30^,^31^,^32^.

## Results

### Generation of mice overexpressing GILZ

**Figure 1:**
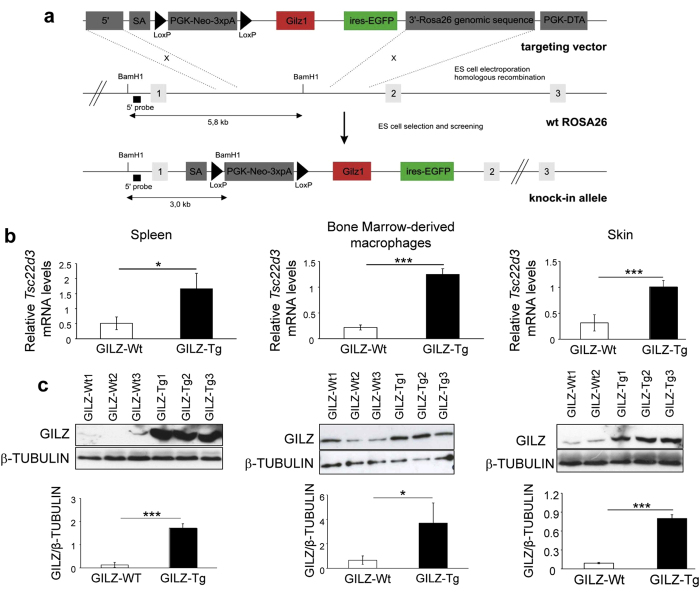
Generation and phenotyping of mice overexpressing GILZ (GILZ-Tg mice). (**a**) Scheme of the transgene construct. Restriction enzyme sites and the location of the 5′ probe used for Southern blot analysis are depicted. The ROSA26 locus was targeted by homologous recombination using the targeting vector (top). At the end, the ROSA26 locus was modified (bottom). (**b**) Relative mRNA levels of *Tsc22d3*/*Gilz1* in the indicated tissues were assessed by RT-QPCR. Mean values ± SD are shown; asterisks denote statistically significant differences relative to controls (Student’s *t* test; n = 4 per genotype; *p < 0.05; ***p < 0.001). (**c**) Representative Western blot showing GILZ protein levels in the spleen, bone marrow-derived macrophages, and skin of GILZ-Wt and GILZ-Tg mice. Tubulin is shown as a loading control. Quantitation of Western blot shows mean values ± SD; asterisks denote statistically significant differences relative to controls (Student’s *t* test; n = 3 per genotype; *p < 0.05; ***p < 0.001).

Using a Gateway-compatible ROSA26 locus targeting vector^33^, we generated conditional GILZ-Tg overexpressing mice by knocking in the mouse *Tsc22d3/Gilz-1* cDNA preceded by a loxP flanked stop cassette under control of the ROSA26 promoter (Fig. 1a). Mice homozygous for the loxP flanked stop cassette and one allele of Nestin-cre, expressing Cre in all cell types, especially in the brain, testis and gut and to a lesser extent in the liver^34^, were generated by crossing. These mice were used in all the experiments and are designated as GILZ-Tg mice. Because of some degree of leakiness of the loxP flanked stop cassette, we used mice without the cassette, but with the same genetic background as the GILZ-Tg mice as controls (GILZ-Wt). The GILZ-Tg mice were viable and showed relative increases of expression of the transgene in a tissue-dependent manner ranging between 3- and 6-fold (Fig. 1b and data not shown). We also detected a significant increase of GILZ at the protein level in various tissues including spleen and bone marrow-derived macrophages (Fig. 1c). In adult skin, the overexpression of GILZ at the mRNA and protein level ranged between 3- and 8-fold and did not cause any obvious histopathological changes in tissue architecture (Fig. 1b,c and Fig. S1).

### GILZ overexpression increases IMQ-induced psoriasis-like skin lesions

**Figure 2:**
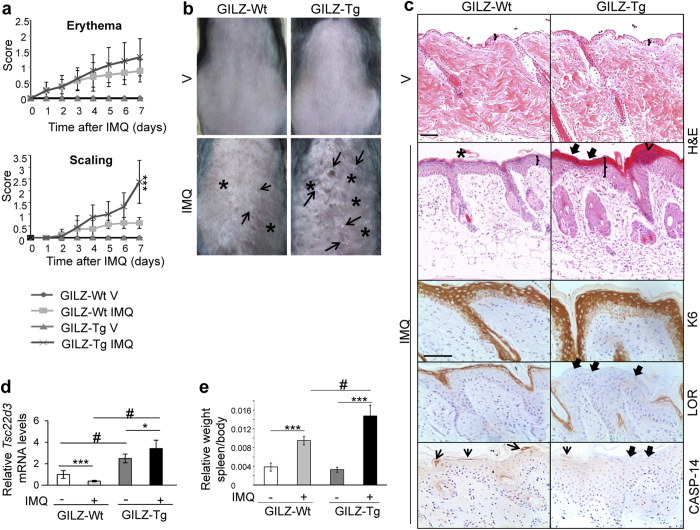
GILZ-Tg mice show increased psoriatic features in response to imiquimod (IMQ). (**a**) Score of the erythema and scaling in GILZ-Wt and GILZ-Tg for the duration of the experiment. V: vehicle; IMQ, imiquimod. (**b**) Macroscopical appearance of GILZ-Wt and GILZ-Tg mice treated with IMQ at day 7. (**c**) Representative Hematoxilin&Eosin (H&E) stained and immunohistochemistry sections of adult mouse skin of IMQ-treated GILZ-Wt and GILZ-Tg mice. Epidermal thickening (brackets), hyperkeratosis (asterisks), abnormal differentiation with parakeratosis (thick arrows), and epidermal infiltrate (Munro-like abscess, arrowhead) are indicated. Immunostaining with keratin (K)6, Loricrin (LOR), and Caspase-14 (CASP-14) antibodies is also shown. Note the increased dermal cellularity in IMQ-treated GILZ-Tg relative to GILZ-Wt mice. Bar: 50 μm. Samples from two independent experiments were assessed (n = 15 GILZ-Wt, n = 16 GILZ-Tg). (**d**) Relative *Tsc22d3*/*Gilz* mRNA levels in GILZ-Wt and GILZ-Tg mouse skin were assessed by RT-QPCR before and after treatment with IMQ at day 7. (**e**) Splenomegaly was determined by the weight ratio of spleen relative to body. (**d**,**e**) Mean values ± SD are shown. Post hoc Tukey test ^#^p < 0.05; ***p < 0.001, n = at least 5 per genotype and treatment. Asterisks: significance between treatments within each genotype; hashes: significance between genotypes in the same treatment group.

To investigate the role of GILZ overexpression in the IMQ model, we topically treated GILZ-Tg and GILZ-Wt mice with Aldara^®^ or control cream for 7d^29^,^30^ (Fig. 2). Erythema (redness) and scaling were scored daily. We found that the response to IMQ was more pronounced in GILZ-Tg relative to GILZ-Wt mice with marked increases in skin alterations from day 4 onwards and significant differences in scaling at d7 (Fig. 2a, approximately 4-fold). Representative images of the increased skin erythema (asterisks) and severe desquamation (arrows) after 7d of IMQ treatment in the GILZ-Tg mice are shown (Fig. 2b).

The histopathology of IMQ-treated mouse skin is characterized by epidermal thickening, abnormal epidermal differentiation and neutrophil infiltration (Munro-like abscess), which closely resemble the human histological picture of the disease^1^,^6^,^29^. IMQ treatment resulted in epidermal thickening in both GILZ-Wt and GILZ-Tg mice relative to untreated animals (Fig. 2c, H&E; brackets), with greater increased epidermal width in IMQ-treated GILZ-Tg mice. Scaling of the skin often reflects abnormal keratinocyte differentiation with retention of the nuclei in the stratum corneum, the outer most layer of the epidermis (parakeratosis). Consistent with the augmented scaling in IMQ-treated GILZ-Tg mice (Fig. 2a), we also detected overall increased parakeratosis (Fig. 2c, H&E; thick arrows). In addition, we also detected the presence of cell infiltrates just beneath the stratum corneum (Fig. 2c, H&E; arrowhead), highly similar to Munro-like abscesses in psoriasis skin lesions.

We assessed the expression of keratin (K)6, which is normally restricted to hair follicles but becomes up-regulated in diseased skin^35^. Immunostaining showed hyperproliferative epidermis positive for K6 in both GILZ-Wt and GILZ-Tg mice (Fig. 2c, K6). The expression of loricrin was confined to the upper differentiated layers of the epidermis in GILZ-Wt while this marker was focally absent in GILZ-Tg mice correlating with augmented parakeratosis^36^ (Fig. 2c, LOR; thick arrows).

One possible mechanism by which nuclei accumulate in the stratum corneum is by impaired expression and/or function of Caspase-14 (CASP-14), a unique non-apoptotic epidermal-specific caspase that is crucial for a functional skin barrier as it mediates the proteolytic processing of pro-filaggrin to make granules of the epidermal granular layer^37^,^38^. In GILZ-Wt skin, CASP-14 was detected in the more differentiated epidermal layers while it was absent in GILZ-Tg skin, correlating with parakeratotic areas (Fig. 2c, CASP-14; arrows and thick arrows, respectively).

Besides these epidermal alterations, psoriasis is characterized by pronounced inflammatory infiltrates mainly consisting of T cells and dendritic cells. Upon IMQ treatment, the dermal cellularity was clearly increased in GILZ-Tg mice relative to GILZ-Wt mice (Fig. 2c, compare dermal infiltrates). Note that the endogenous *Tsc22d3* mRNA levels decreased by approximately 60% in IMQ-treated GILZ-Wt skin (Fig. 2d), in agreement with the previously reported *Tsc22d3* down-regulation in various inflammatory settings^14^. In contrast, *Tsc22d3* mRNA levels remained high before and after the IMQ treatment in GILZ-Tg skin (Fig. 2d).

### Augmented susceptibility of GILZ-Tg mice to IMQ-induced psoriasis is not due to increased circulating inflammatory cytokines

It has been demonstrated that besides the cutaneous phenotype, topical IMQ induced systemic effects including increased circulating cytokines and splenomegaly^29^,^30^,^32^. Consistent with these published data, we detected an increase in the relative spleen/body ratio in control mice and this splenomegaly was further increased in GILZ-Tg mice (Fig. 2e, 2-fold *vs* 3-fold induction, respectively).

**Figure 3:**
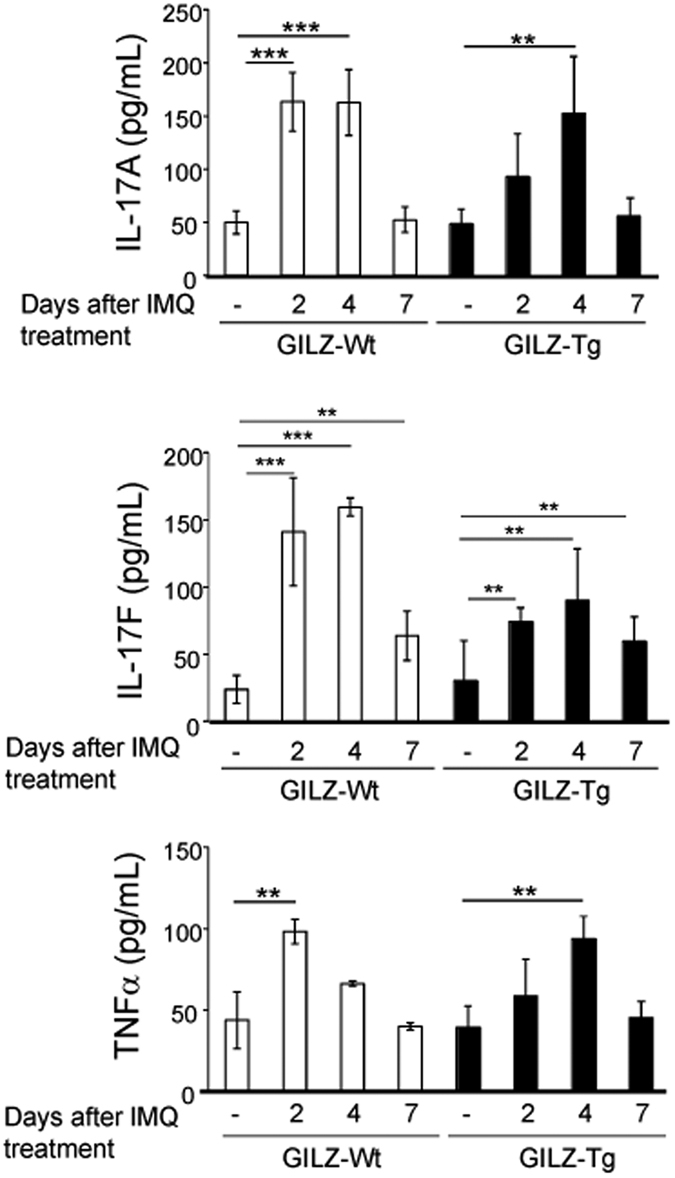
Augmented susceptibility of GILZ-Tg mice to IMQ-induced psoriasis is not due to increased circulating inflammatory cytokines. Serum levels of circulating cytokines IL-17A, IL-17F, and TNFα were assessed in GILZ-Tg and GILZ-Wt mice treated with IMQ for 0, 2, 4, and 7 d. Mean values ± SD are shown. Post hoc Tukey test **p < 0.01; ***p < 0.001; n = at least 3 per genotype and treatment. Asterisks: significance between treatments within each genotype; no statistical significance was detected between genotypes in the same treatment group.

We also assessed the serum levels of IL-17A, IL-17F, and TNFα in GILZ-Tg and GILZ-Wt mice treated with IMQ for 0, 2, 4, and 7 d. In control mice, and according to our previous reports^32^ all these cytokines were induced by IMQ (2–7-fold) with a peak of expression at 2–4 d after treatment (Fig. 3). The levels of these circulating cytokines increased to a similar extent in GILZ-Tg mice although with small variations in the kinetics of induction (Fig. 3). These data suggest that the augmented susceptibility of GILZ-Tg mice to IMQ-induced psoriasis is due to differences in the cutaneous response rather than differences in the systemic response.

### Increased up-regulation of Th17-dependent cytokines in GILZ-Tg mouse skin

**Figure 4:**
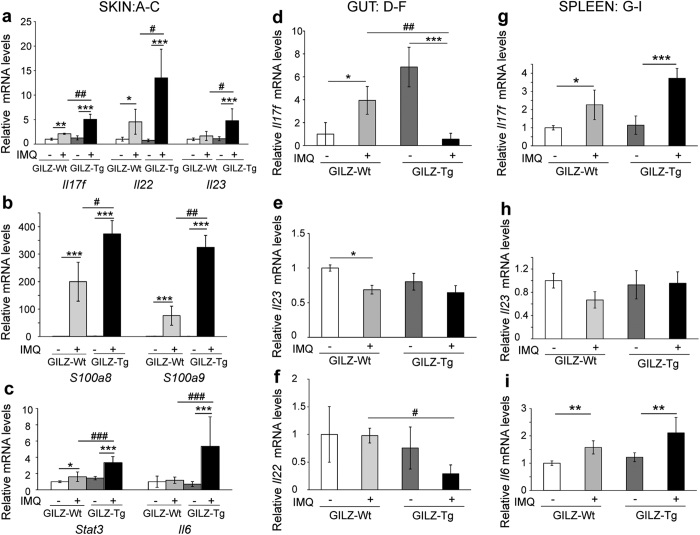
Increased up-regulation of inflammatory mediators in IMQ-treated GILZ-Tg relative to GILZ-Wt mouse skin. Relative mRNA levels of the indicated genes were assessed by RT-QPCR in skin (**a**–**c**), gut (**d**–**f**), and spleen (**g**–**i**) collected from the same individuals after IMQ treatment (7d). Mean values ± SD are shown. Post hoc Tukey test *, ^#^p < 0.05; **, ^##^p < 0.01; ***, ^###^p < 0.001; n = at least 5 per genotype and treatment. Asterisks: significance between treatments within each genotype; hashes: significance between genotypes in the same treatment group.

Next, we assessed the cutaneous relative mRNA levels of several cytokines of the IL-23–Th17 axis as well as other inflammatory markers known to be up-regulated in human and mouse psoriatic skin^6^,^30^. We detected significant up-regulation of *Il-17f* and *Il-22* mRNA levels (2.5- and 4-fold, respectively) and a trend of *Il-23* induction (although it did not reach significance) in IMQ-treated *vs* untreated GILZ-Wt skin (Fig. 4a). The lack of consistent *Il-23* up-regulation was likely due to kinetics and/or individual variability. Importantly, all these cytokines were significantly more up-regulated in lesioned GILZ-Tg skin relative to controls (Fig. 4a, 4- to 12-fold). The calcium-binding proteins S100A8/A9 are the most up-regulated proteins in lesional psoriatic skin, and are expressed in keratinocytes and immune cells such as neutrophils^39^. IL6 and STAT3 are also induced in human patients and mouse models of psoriasis^1^. We detected statistically significant increases of *S100a8, S100a9*, an*d Stat3* in IMQ-treated GILZ-Wt mice and notably, the levels of these markers as well as *Il6* were further augmented in GILZ-Tg treated mice (Fig. 4b,c; 1.5- to 5-fold higher than in Gilz-Wt). Our data thus show that transgenic mice overexpressing GILZ treated with IMQ exhibit increased psoriasiform-like features by histological and molecular criteria.

### In IMQ-treated GILZ-Tg mice, the pro-inflammatory actions of overexpressed GILZ occur specifically in skin but not in other tissues

We wondered whether GILZ overexpression was pro-inflammatory in other tissues after topical IMQ treatment as several organs are also affected by this protocol including the spleen^29^ (Fig. 2e) and the intestine, recently claimed as an important amplifying organ in the IMQ model^29^,^32^. We thus collected the spleen and intestines of GILZ-Wt and GILZ-Tg mice before and after IMQ treatment to assess the expression of Th-17 dependent cytokines.

In the gut of transgenic mice, the relative mRNA levels of *Il-17f, Il-22,* and *Il-23* after IMQ treatment were either down-regulated or unchanged relative to IMQ-treated controls (Fig. 4d–f). However, in the spleen, *Il-17f* and *Il-6* mRNA levels by IMQ showed an increased trend of up-regulation in transgenics relative to controls although without statistical significance (Fig. 4g–i); in this tissue, *Il-23* mRNA levels showed no change among treatments or genotypes.

These data demonstrate that the cytokine profile in the skin of IMQ-treated GILZ-Tg mice was different to that of intestine or spleen and suggests that in this disease model, GILZ may act as pro-inflammatory specifically in the skin.

### IMQ-induced psoriasis in GILZ-Tg mice involves cutaneous activation of TGF-β1/SMAD2/3

**Figure 5:**
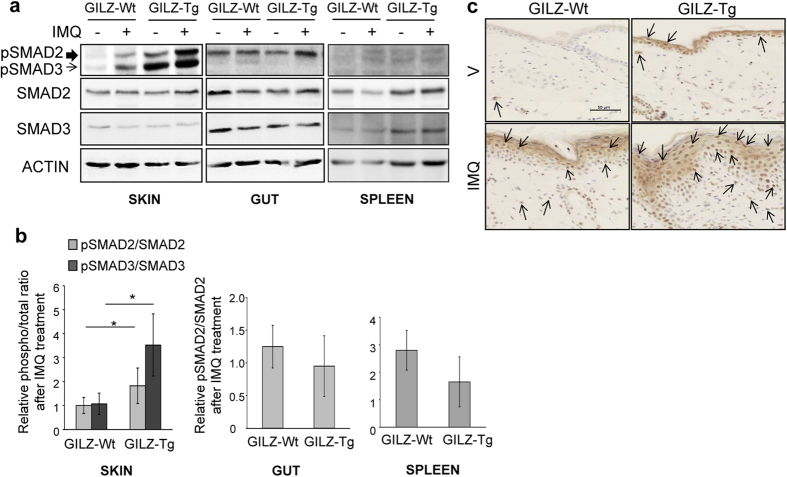
TGF-β1 signaling mediator SMAD2/SMAD3 is overactivated by IMQ treatment in GILZ-Tg mouse skin but not in other tissues. Representative immunoblotting showing the expression of total and phospho(p)-SMAD2/3 in skin (left panels), gut (middle panels), and spleen (right panels) collected from the same individuals (GILZ-Wt and GILZ-Tg) before and after IMQ treatment (7d). Actin is shown as a loading control. (**b**) Quantitation of immunoblotting shows mean values ± SD; asterisks denote statistically significant differences relative to controls (Student’s *t* test; n = 31; *p < 0.05). (**c**) Immunostaining with p-SMAD2/3 antibody demonstrates induction of the TGF-β1 signaling pathway in the epidermis and dermis of IMQ-treated GILZ-Tg and GILZ-Wt mice. Bar: 50 μm. (**a**–**c**) Samples from two independent experiments were assessed (n = 15 GILZ-Wt, n = 16 GILZ-Tg).

It has been reported that GILZ enhances TGF-β1 signaling by binding to and promoting SMAD2/3 phosphorylation and activation of FoxP3 expression^26^. Also, mice overexpressing TGF-β1 in keratinocytes developed phenotypes and molecular alterations similar to human psoriasis, via a SMAD2/3-dependent mechanism^7^,^40^. To assess whether GILZ overexpression correlates with an augmented TGF-β1-mediated signaling, we determined the expression of total and phosphorylated (p)-SMAD2/3. Our data show constitutive increase of active SMAD2/3 ratio in GILZ-Tg relative to GILZ-Wt untreated skin as well as an augmented SMAD2/3 activation after IMQ treatment (Fig. 5a,b; left panels). Consistent with these findings, we detected p-SMAD2/3 in the skin of untreated and IMQ-treated GILZ-Tg mice by immunolocalization (Fig. 5c). It is worth noting that p-SMAD2/3 was found in the epidermal and dermal compartments although its activation in IMQ-treated GILZ-Tg mice was more robust in epidermal keratinocytes.

Importantly, activation of TGF-β1 signaling occurred specifically in skin but not in gut or spleen of GILZ-Tg and GILZ-Wt mice (Fig. 5a,b; middle and right panels). We also assessed possible changes in *Tgfb1* mRNA levels in skin and spleen but found no differences among treatments or genotypes (Fig. S2), suggesting that GILZ overexpression alters TGF-β1 activity but not its expression levels.

### Contribution of immune cells and keratinocytes to the increased psoriasiform features in GILZ-Tg mice

We next tried to assess whether the more severe psoriasis-like phenotype found in IMQ-treated GILZ-Tg mice was due to the contribution of skin infiltrating immune cells, keratinocytes, or both. As an initial approach, we assessed the composition of the immune infiltrates in the skin of GILZ-Tg and GILZ-Wt mice treated with vehicle or IMQ for 7 d by FACS analysis. Despite minor differences, there were no significant changes in the neutrophil, dendritic cells, or T cell populations among genotypes (Fig. S3).

**Figure 6:**
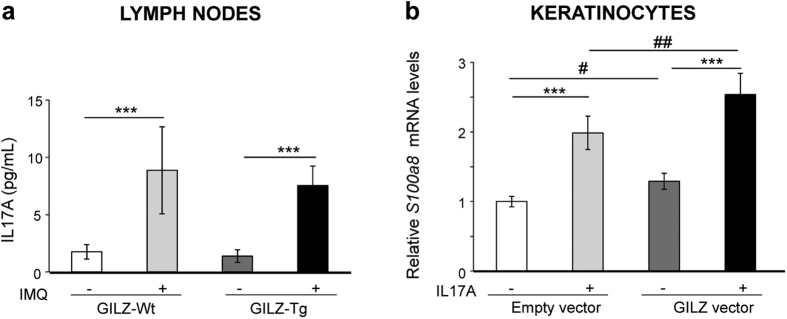
GILZ overexpression in keratinocytes correlates with a significant enhanced response to IL-17A. (**a**) Brachial lymph nodes were isolated from either vehicle- or IMQ-treated GILZ-Tg and GILZ-Wt mice (n = 3 mice per genotype and treatment) after 7d. Cells were then re-stimulated with vehicle or IMQ for 3 d, and IL-17A levels were measured in the supernatant. (**b**) Cultured keratinocytes were transfected with either an empty vector or GILZ cDNA containing plasmid and the expression of *S100a8*. mRNA levels was assessed after IL-17A treatment (100 ng/ml) for 24 h. n = at least 6 independent replicates per transfection and treatment. Mean values ± SD are shown. Post hoc Tukey test ^#^p < 0.05; ^##^p < 0.01; ***p < 0.001. Asterisks: significance between treatments within each genotype; hashes: significance between genotypes in the same treatment group.

We then examined more directly whether immune cells and keratinocytes showed a differential response to inflammation *in vitro*. First, we isolated lymph nodes from GILZ-Tg and GILZ-Wt mice that had been treated with cream or IMQ for 7 d, re-stimulated the cells with IMQ for 3 d, and checked IL-17A in the supernatant (Fig. 6a). We detected a significant but similar induction of IL-17A in both control and GILZ-Tg cells (5- and 5.5-fold, respectively) indicating that IMQ induces the Th17-mediated response in lymph nodes equally in both genotypes (Fig. 6a).

We also assessed the effects of IL-17A on the expression of the psoriatic marker *S100a8* in cultured keratinocytes that were transiently transfected with either an empty vector or GILZ cDNA containing plasmid. Importantly, both basal and IL-17A-induced *S100a8* mRNA levels were significantly higher (approximately 30%) in GILZ-transfected keratinocytes relative to controls (Fig. 6b). Collectively, these data indicate that despite IMQ triggers a similar enhanced Th17-mediated response in immune cells from control and GILZ-Tg mice, it results in higher induction of the marker of psoriasis *S100a8* in GILZ overexpressing keratinocytes (Fig. 6).

## Discussion

The generation and characterization of a mouse model with generalized overexpression of GILZ allowed us to demonstrate that although GILZ is considered an anti-inflammatory therapeutic protein in different experimental models, GILZ-Tg mice showed increased susceptibility to IMQ-induced psoriasis by histological and molecular criteria.

The score of skin lesions showed significant differences in scaling but not in erythema in GILZ-Tg relative to GILZ-Wt mice at d7 (Fig. 2a). However, there was a tendency towards increased erythema in GILZ-Tg *vs* GILZ-WT mice from d4 onwards, which was not statistically significant likely due to masking of the reddening by the severe desquamation in GILZ-Tg mice (Fig. 2a,b).

The histopathology of IMQ-treated GILZ-Tg mouse skin also featured more pronounced epidermal thickening, parakeratosis, and neutrophil infiltration relative to controls (Fig. 2). Importantly, we found that the severe parakeratosis in GILZ-Tg mice correlated with the absence of CASP-14, a caspase whose proteolytic role is required for normal epidermal differentiation and that is usually confined to the upper epidermal layers (Fig. 2c). However, the lack of CASP-14 is not likely to cause the development of parakeratotic plaques but rather contribute to the formation of a defective barrier and, thus, to the aggravation of psoriatic lesions, as CASP-14 knock-out mice did not show spontaneous parakeratosis^38^ but developed increased parakeratosis after IMQ treatment^40^. How GILZ modulates CASP-14 is currently unknown but given its restricted expression to differentiating keratinocytes, it is plausible that the observed CASP-14 down-regulation in GILZ-Tg skin reflects alterations in other transcription factors regulating epidermal differentiation^38^. Also, the focal absence of CASP-14 in GILZ-Tg skin lesions is consistent with previous work reporting that CASP-14 was reduced in lesional psoriatic skin likely due to the action of T-helper type 2 cytokines^38^,^41^,^42^. It is currently unknown whether the increased action of Th17-dependent cytokines in GILZ-Tg mouse skin also plays a causative role in down-regulating CASP-14 function.

One major question is whether these severe psoriasis-like features in GILZ-Tg mice are due to systemic and/or cutaneous local responses to IMQ. According to previous reports^32^, our data showed increased production of circulating cytokines after IMQ treatment but there were no differences among genotypes (Fig. 3) ruling out that a differential systemic response contributes to the severity of the GILZ-Tg skin lesions.

It is also plausible that both skin infiltrating immune cells and keratinocytes may contribute to the more severe cutaneous phenotype of IMQ-treated GILZ-Tg mice. Our data showed no major differences in the composition of skin neutrophil or T cell infiltrates of GILZ-Tg and GILZ-Wt mice nor in the response of isolated lymphoid cells to IMQ (Figs S3 and 6a). However, we demonstrated that the overexpression of GILZ in cultured keratinocytes induced a significantly enhanced up-regulation in the mRNA levels of the psoriatic marker *S100a8* in response to IL-17A (Fig. 6b). Overall, our data indicate that the pro-inflammatory actions of GILZ are specific to the skin as neither the intestine nor spleen showed increased levels of Th17-dependent cytokines relative to controls (Fig. 4). Also, these pro-inflammatory effects of GILZ seem to be enhanced in keratinocytes in a cell-type-specific manner likely contributing to the increased psoriasiform features in GILZ-Tg mice.

Given that *Tsc22d3* was strongly down-regulated in IMQ-treated GILZ-Wt but not in GILZ-Tg skin (Fig. 2d), it is also possible to speculate that GILZ down-regulation is required for resolution of the inflammation and that the continuous expression of GILZ in transgenic skin exerts paradoxical pro-inflammatory actions. Importantly, it was previously reported that *Tsc22d3* was also down-regulated by 2.6-fold in the skin of psoriatic lesions *vs* non-lesional matched biopsies^43^. Thus, it is feasible that the observed pro-inflammatory effects are the consequence of high levels of exogenous GILZ instead of physiological levels, indicating that GILZ activity must be tightly controlled for skin homeostasis.

It is also important that in this model, the pathological actions of GILZ correlated with skin-specific TGF-β1/SMAD2/3 activation (Fig. 5). Thus, caution is required when considering the use of GILZ as an alternative treatment for psoriasis since high doses and/or long treatments may also have adverse effects. It is tempting to hypothesize that the observed insensitivity and/or resistance of a subset of psoriatic patients treated with GCs may be due to GILZ overexpression eventually leading to increased p-SMAD2/3, which would result in aggravation of skin lesions.

Our results are seemingly contradictory to the fact that GILZ-deficient mice treated with IMQ exhibited increased Th17-mediated inflammation^25^. Importantly, untreated GILZ-deficient mice already showed spontaneously increased production of IL-17 and IL-22 by lymph node cells *in vivo*^25^. While these results demonstrate a key role for GILZ in regulating Th-17 dependent cytokines by T cells and dendritic cells, it is unclear whether this basal inflammation contributes to the increased susceptibility of GILZ-deficient mice to IMQ. In contrast, in the absence of treatment, GILZ-Tg and GILZ-Wt mouse skin exhibited similar expression of these cytokines with significant increases occurring only in response to IMQ (Fig. 4a). Also, we have characterized the skin alterations of mice overexpressing GILZ after IMQ treatment while the report by Morand and colleagues did not specifically address the epidermal phenotype^25^. The apparent discrepancies between the KO results and our results highlight that the use of complementary approaches such as GILZ-deficient *vs* GILZ-Tg mice may be necessary to provide a full understanding of the true biology behind a protein. Taken together the results of the gain and loss of function models indicate that GILZ levels must be tightly regulated to effectively reduce inflammation, with too much or too little having pathological consequences.

## Materials and Methods

### Animals

#### Generation of GILZ transgenic mice

The generation of GILZ-Tg mice is described in the Results section. The construct contained the mouse *Tsc22d3/Gilz-1* cDNA preceded by a loxP flanked stop cassette under control of the ROSA26 promoter, and followed by an IRES-EGFP cDNA. After identifying correctly targeted embryonic stem cell clones by Southern blotting, chimeric mice that transmitted the transgene to their offspring were generated. The experimental mice were homozygous for the transgenic construct and had one Nestin-cre allele and are designated as GILZ-Tg mice. As controls, we used mice without the cassette (GILZ-Wt), but with exactly the same genetic background (C57BL/6).

Mice were maintained at 12 light/12 dark cycle, caged in groups (4–6 per cage) under SPF conditions, and having access to *ad libitum* food and water. All the animal protocols were approved by the ethical committee of the Faculty of Sciences at Ghent University, and followed the European guidelines and regulations.

#### Imiquimod-induced psoriasis-like skin lesions

Female GILZ-Wt and GILZ-Tg mice (8–12 weeks) were used for the IMQ-induced psoriasis mouse model. Two days after shaving, Aldara^®^ (5% IMQ, 3 M Pharmaceuticals; 62.5 mg) or control cream (Ava, Fagron NV) was topically applied on mouse dorsal skin daily for 7 consecutive days. Macroscopic parameters such as skin erythema and scaling were daily scored independently on a scale from 0 to 4: 0, none; 1, slight; 2, moderate; 3, marked; 4, very marked. On day 7, a section of dorsal skin and gut was collected, snap-frozen and stored at −80 °C until use or fixed in 70% ethanol for histopathological analysis. Spleens were collected and weight was expressed relative to mouse body weight. The total number of animals used was 31 (15 GILZ-Wt, 16 GILZ-Tg; two independent experiments).

#### Antibodies

Polyclonal antibodies against pSMAD2/3 Ser 465/467 (3108 P), SMAD2 (5339 P), and SMAD3 (9523 P) were from Cell Signaling Technology, Inc. Actin (A2066, Sigma-Aldrich, St. Louis, MO) was used as protein loading control. Secondary peroxidase-conjugated anti-rabbit antibodies were from GE HealthCare. The GILZ antibody was purchased from eBioscience (14-4033).

For immunostaining, rabbit polyclonal antibodies against K6 (PRB-169P) and loricrin (PRB-145P) were from Covance, Berkeley, CA, and Caspase-14 (sc-5628) and p-SMAD2/3 (sc-11769-R) were from Santa Cruz, CA. Secondary biotin-conjugated anti-rabbit or anti-mouse antibodies (Jackson ImmunoResearch, West Grove, PA) were used.

#### Histology and immunohistochemistry analysis

Mouse dorsal skins (n = 15 GILZ-Wt, n = 16 GILZ-Tg) were dissected and fixed in 70% ethanol for two days, then in paraffin. 4-μm thick sections were used for hematoxylin/eosin (H&E) staining and immunohistochemical analysis. After incubation with primary and secondary biotin-conjugated antibodies immunoreactivity was revealed using the ABC kit Vectastain (PK-6100; Vector Labs) and the sections were counterstained with hematoxylin. All skin samples were evaluated for histopathological analysis.

#### RNA isolation and quantitative RT-PCR

Total RNA was isolated from mouse dorsal skin using Trizol (Life Technologies). After RNA isolation, DNase treatment of the samples was done according to the manufacturer’s instructions (Thermo Scientific). Subsequently, samples were reverse transcribed to make cDNA using RevertAid H Minus Reverse Transcriptase and using oligo dT primers (Thermo Scientific). Quantitative real-time PCR was performed using specific primers and FastStart Universal SYBR Green Master ROX (Roche) on Applied Biosystems 7500 Fast real time PCR machine. *Hprt1, Ubc* and *Rpl* were used as housekeeping genes. At least four to five biological replicates were used per genotype and treatment. The sequences of primers used are as follows: *Hprt*1 (Forward: 5′-AGTGTTGGATACAGGCCAGAC-3′, Reverse: 5′-CGTGATTCAAATCCCTGAAGT-3′), *Ubc* (Forward: 5′-AGGTCAAACAGGAAGACAGACGTA-3′, Reverse: 5′-TCACACCCAAGAACAAGCACA-3′), *Rpl* (Forward: 5′-CCTGCTGCTCTCAAGGTT-3′, Reverse: 5′-TGGTTGTCACTGCCTCGTACTT-3′), *S100a8* (Forward: 5′-AAATCACCATGCCCTCTACAAG-3′, Reverse: 5′-CCCACTTTTATCACCATCGCAA-3′), *S100a9* (Forward: 5′-ATACTCTAGGAAGGAAGGACACC-3′, Reverse: 5′-TCCATGATGTCATTTATGAGGGC-3′), *Il-17f* (Forward: 5′-CCCAGGGCTGTTCTAATTCCTT-3′, Reverse: 5′-GACACAGGTGCAGCCACCTTT-3′), *Stat3* (Forward: 5′-AGCTGGACACACGCTACCT-3′, Reverse: 5′-AGGAATCGGCTATATTGCTGGT-3′), *Il-22* (Forward: 5′-TCAGTGCTAAGGATCAGTGCT-3′, Reverse: 5′-TGATTGCTGAGTTTGGTCAGG-3′), *Il-23a* (Forward: 5′- AAAATAATGTGCCCCGTATCCAG-3′, Reverse: 5′- GCTCCCCTTTGAAGATGTCAG-3′), *Il-6* (Forward: 5′- TAGTCCTTCCTACCCCAATTTCC-3′, Reverse: 5′- TTGGTCCTTAGCCACTCCTTC-3′), *Tsc22d3/Gilz1* (Forward: 5′- CGGTCTATCAGCTGCACAATTT-3′, Reverse: 5′- ACATCCCCTCCAAGCAGAGA-3′).

#### Western blotting

Whole-cell protein extracts from mouse dorsal skin and cultured keratinocytes were prepared as previously described^44^. Samples were boiled in Laemmli buffer, separated on 10% SDS-PAGE, and transferred onto nitrocellulose membranes (Hybond ECL, GE Healthcare). Gel loading control was done by Ponceau S staining on the membranes after transfer. Membranes were blocked and incubated with specific polyclonal primary antibodies followed by incubation with peroxidase-conjugated anti-rabbit secondary antibodies. The immunoreactive bands were analyzed using ECL2 (Thermo Scientific) with the ImageQuant 4000 Biomolecular Imager (GE Healthcare). Protein band density was determined using Image J software. Experiments were performed with a minimum of three biological replicates.

#### Determination of serum cytokine levels

Serum samples were assayed for IL-17A, IL-17F, and TNFα using Luminex technology (BioPlex, Bio-Rad) in accordance with the manufacturer’s protocol.

#### Isolation of lymph nodes, treatments, and determination of IL-17A levels

Ear-draining (brachial) lymph nodes were isolated at d7 from GILZ-Tg and GILZ-Wt mice treated with Aldara or vehicle (Ava cream). Lymph nodes were minced through a 70 μm cell strainer (BD Falcon) to obtain single cell suspensions which were re-stimulated with 10 μg/ml Aldara or vehicle for 3 d. Cells were cultured in RPMI-1640L medium supplemented with Glutamax, 10% fetal calf serum, gentamycin and β-mercaptoethanol (Gibco; Life Technologies) IL-17A cytokine production was measured in the supernatant of cultured cells using Luminex technology (BioPlex; Bio-Rad) in accordance with the manufacturer’s protocol.

#### Keratinocyte cells, transfection and treatments

Keratinocytes were isolated from 8-week-old C57BL6/DBA female mouse dorsal skin and immortal keratinocyte cell lines were generated and cultured as previously described^28^. Keratinocytes were seeded in a 6-well plate to a confluence of about 80% 24 h prior to transfection. Empty expression vector cassette pCDNA4 was used as a negative control. Keratinocytes were transiently transfected with pEGFP-C1-Gilz1 plasmid (500 ng) using Lipofectamine 2000 Reagent (Invitrogen) according to the manufacturer’s protocol. Five hours after transfection, medium was replaced and cells were treated with recombinant human IL-17A (R&D Systems) at a concentration of 100 ng/ml for 24 h.

#### Statistical Analysis

Experimental data were analyzed using Microsoft Excel and IBM SPSS Statistics 23 software. In all graphs, data are expressed as the mean value ± SD. When statistical analysis was performed with relative values, data were first subjected to logarithmic transformation. Prior to parametric testing, the Levene’s test was used to determine whether samples within groups had equal variance. For comparisons between two experimental groups, we used the Student’s unpaired two-tailed t-test. For comparisons among more than two experimental groups, we used the one-way ANOVA which if statistically significant was followed by a post hoc Tukey multiple comparison test. P values less than 0.05 were considered statistically significant.

## Additional Information

**How to cite this article**: Carceller, E. *et al*. Overexpression of Glucocorticoid-induced Leucine Zipper (GILZ) increases susceptibility to Imiquimod-induced psoriasis and involves cutaneous activation of TGF-β1. *Sci. Rep.* **6**, 38825; doi: 10.1038/srep38825 (2016).

**Publisher's note:** Springer Nature remains neutral with regard to jurisdictional claims in published maps and institutional affiliations.

## Supplementary Material

Supplementary Information
