# Supplementary material for: Overexpression of Glucocorticoid-induced Leucine Zipper (GILZ) increases susceptibility to Imiquimod-induced psoriasis and involves cutaneous activation of TGF-β1
Source: Sci Rep. 2016 Dec 9;6:38825. doi: 10.1038/srep38825 (PMC5146970; doi:10.1038/srep38825)
Supplement: Supplementary Information [file srep38825-s1.pdf]

Overexpression of Glucocorticoid-induced Leucine Zipper (GILZ) increases susceptibility to Imiquimod-induced psoriasis and involves cutaneous activation of TGF-β1

Elena Carceller[1, #], Marlies Ballegeer[2, 3, #], Julie Deckers[2,3,4,5], Carlo Riccardi[4], Stefano Bruscoli[4], Tino Hochepied[2,3], Claude Libert[2, 3, *], and Paloma Pérez[1, *]

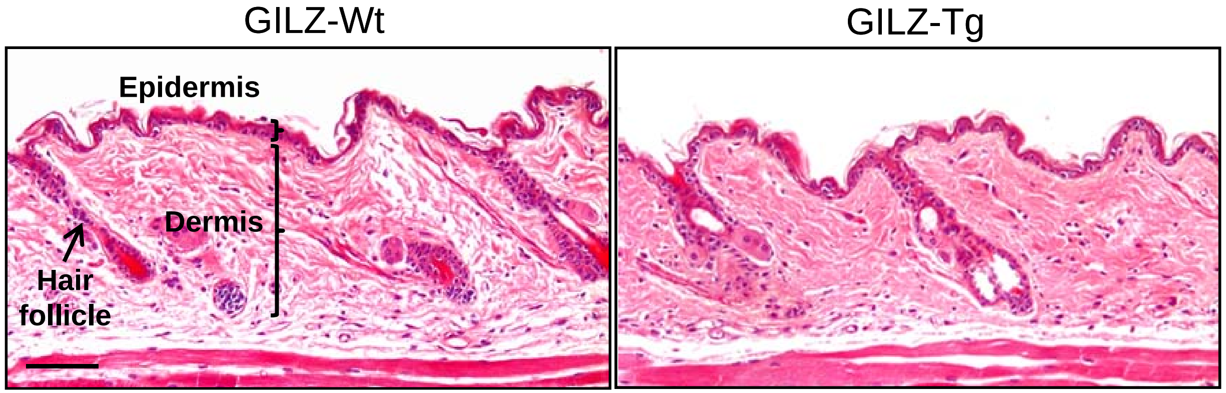

Figure S1. Normal skin architecture of GILZ-Tg mice.
Representative Hematoxilin&Eosin stained section of adult mouse GILZ-Wt and GILZ-Tg skin showing no differences in tissue architecture (n=4 per genotype). Bar: 50 μm.

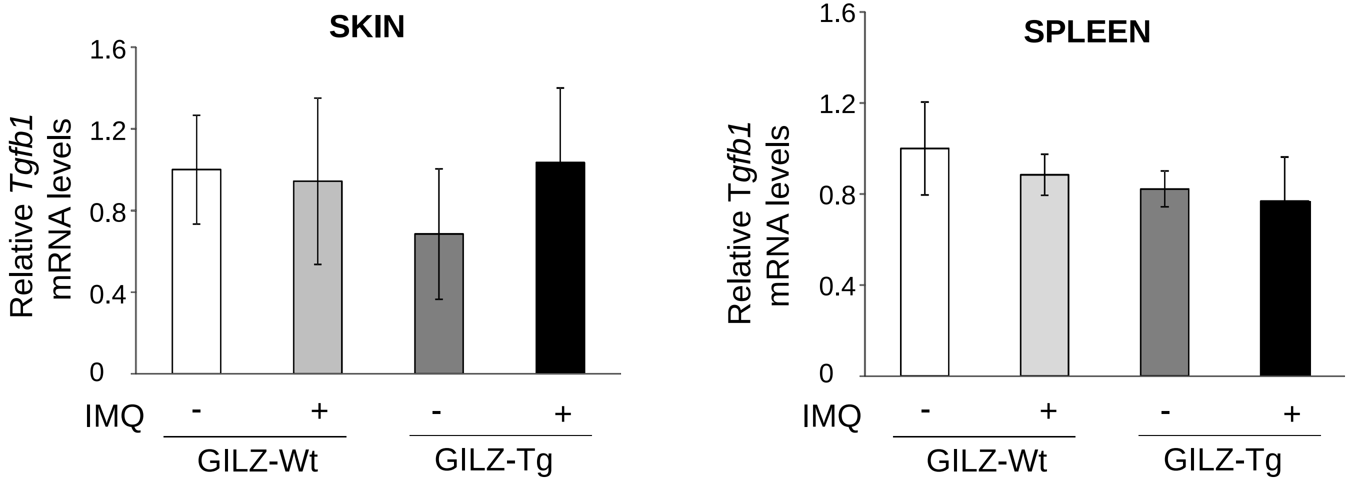

Figure S2. Normal *Tgfb1* mRNA levels in skin and spleen of GILZ-Tg mice.
Relative *Tgfb1* mRNA levels were assessed in skin and spleen of GILZ-Wt and GILZ-Tg mice (n=6 per genotype and treatment). Mean values ± SD are shown. No significant changes were found among genotypes or treatments.

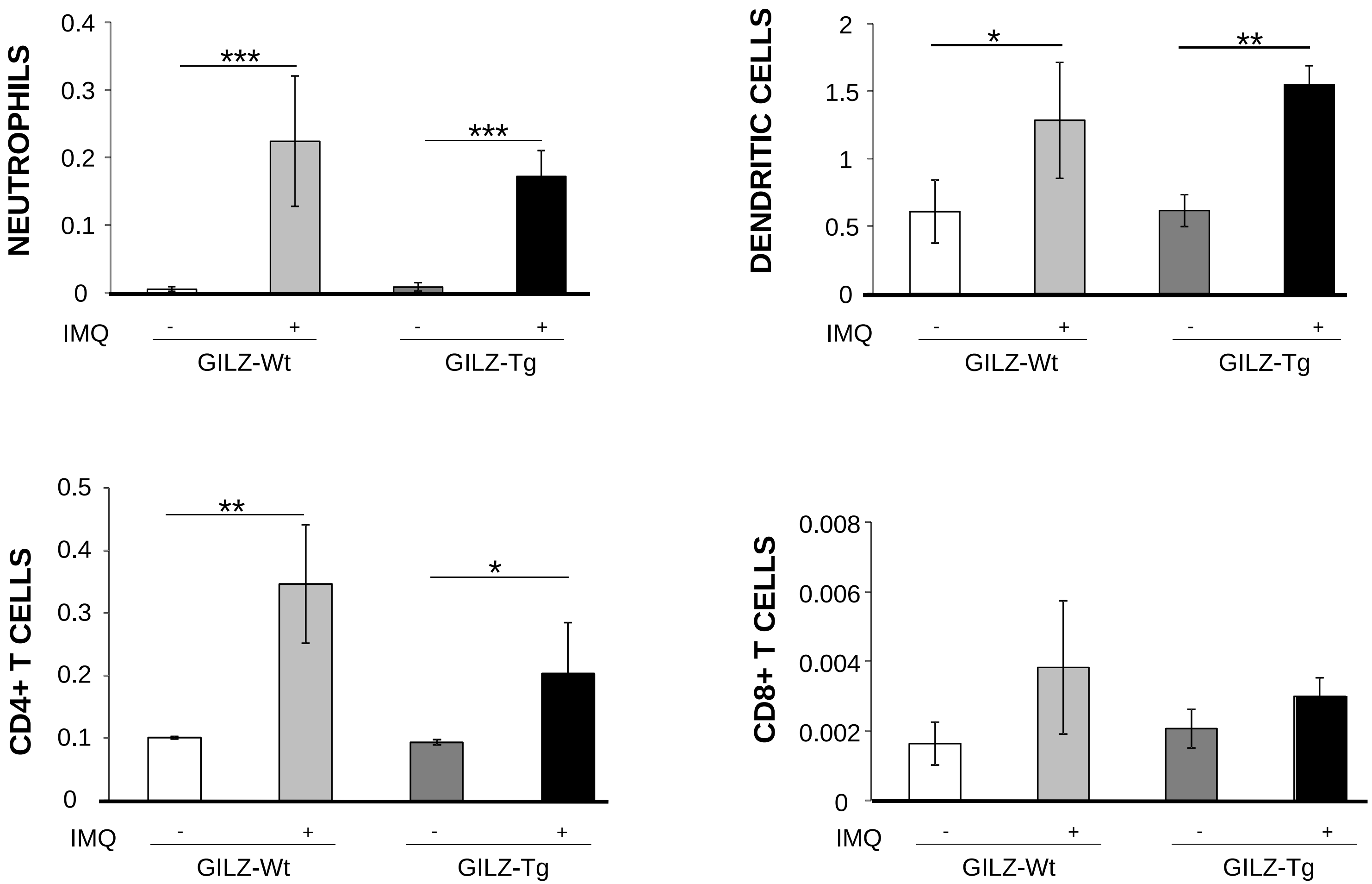

Figure S3. Composition of cutaneous infiltrating immune cells in GILZ-Tg and GILZ-Wt mice. GILZ-Tg and GILZ-Wt mouse ear skin were treated with vehicle or IMQ for 7 d and the composition of the immune infiltrates was determined by FACS analysis. Mean values ± SD are shown. Post hoc Tukey test *, $p<0.05$; **, $p<0.01$; ***, $p<0.001$; n= 4 per genotype and treatment. No significant changes were found between genotypes in the same treatment group.

SUPPLEMMENTARY MATERIALS AND METHODS

Primers

The sequence of primers for *Tgfb1*: Forward: 5'- CTCCCGTGGCTTCTAGTGC-3', Reverse: 5'- GCCTTAGTTTGGACAGGATCTG-3'.

FACS Analysis

Single cell suspensions were prepared out of ear skin. Briefly, whole skin samples were digested overnight at 4°C with Dispase II (Roche, 0.2-0.4 mg/ml). The next day, the samples were homogenized using a scalpel and digested with DNase (Roche, 0.01U/µl) and Collagenase IV (Worthington CLS4, 20mg/ml) for 3 times 30 min at 37°C with vigorous resuspension in between. After filtering with a 100 µM cell strainer (BD Falcon), cell pellets were resuspended in PBS. Next, cells suspensions were stained at 4°C in the dark with the following antibodies: anti-CD45 (PerCp-Cy5.5), anti-Siglec-F (PE), anti-CD3 (Pe-Cy5), anti-CD19 (PE-Cy5), anti-CD11c (PE-Cy7), anti-Ly-6G (AF700), anti-MHC-II (APC-Cy7), anti-CD4 (PE-eFluor610), anti-CD8 (eFluro450). The first step of the staining was done in PBS for 45 min at 4°C, the second step in PBS for 30 min on ice. Data were acquired on a cell analyzer (LSRFortessa 4 laser; BD Biosciences) and analyzed using FlowJo software (Tree Star).
